# Acquired radioresistance in EMT6 mouse mammary carcinoma cell line is mediated by CTLA-4 and PD-1 through JAK/STAT/PI3K pathway

**DOI:** 10.1038/s41598-023-29925-x

**Published:** 2023-02-22

**Authors:** Nur Fatihah Ronny Sham, Narimah Abdul Hamid Hasani, Nurhaslina Hasan, Muhammad Khalis Abdul Karim, Syed Baharom Syed Ahmad Fuad, Harissa Husainy Hasbullah, Mohammad Johari Ibahim

**Affiliations:** 1 Faculty of Medicine, Jalan Hospital, Universiti Teknologi MARA, Selangor Branch, Sungai Buloh Campus, 47000 Sungai Buloh, Selangor Malaysia; 2 Faculty of Dentistry, Jalan Hospital, Universiti Teknologi MARA, Selangor Branch, Sungai Buloh Campus, 47000 Sungai Buloh, Selangor Malaysia; 3 Faculty of Science, Universiti Putra Malaysia, 43400 Serdang, Selangor Malaysia

**Keywords:** Cancer, Cancer models, Tumour biomarkers, Molecular medicine

## Abstract

Cancer recurrence is often associated with the acquisition of radioresistance by cancer tissues due to failure in radiotherapy. The underlying mechanism leading to the development of acquired radioresistance in the EMT6 mouse mammary carcinoma cell line and the potential pathway involved was investigated by comparing differential gene expressions between parental and acquired radioresistance cells. EMT6 cell line was exposed to 2 Gy/per cycle of gamma-ray and the survival fraction between EMT6-treated and parental cells was compared. EMT6^RR_MJI^ (acquired radioresistance) cells was developed after 8 cycles of fractionated irradiation. The development of EMT6^RR_MJI^ cells was confirmed with further irradiation at different doses of gamma-ray, and both the survival fraction and migration rates were measured. Higher survival fraction and migration rates were obtained in EMT6^RR_MJI^ cells after exposure to 4 Gy and 8 Gy gamma-ray irradiations compared to their parental cells. Gene expression between EMT6^RR_MJI^ and parental cells was compared, and 16 genes identified to possess more than tenfold changes were selected and validated using RT-PCR. Out of these genes, 5 were significantly up-regulated i.e., IL-6, PDL-1, AXL, GAS6 and APCDD1. Based on pathway analysis software, the development of acquired radioresistance in EMT6^RR_MJI^ was hypothesized through JAK/STAT/PI3K pathway. Presently, CTLA-4 and PD-1 were determined to be associated with JAK/STAT/PI3K pathway, where both their expressions were significantly increased in EMT6^RR_MJI^ compared to parental cells in the 1st, 4th and 8th cycle of radiation. As a conclusion, the current findings provided a mechanistic platform for the development of acquired radioresistance in EMT6^RR_MJI^ through overexpression of CTLA-4 and PD-1, and novel knowledge on therapeutic targets for recurrent radioresistant cancers.

## Introduction

Breast cancer is one of the leading causes of mortality among women with an estimated 2.1 million cases worldwide^1^. The standard treatments for breast cancer are usually a combination of surgery, radiation, chemotherapy, and targeted therapy^2^. The main role of adjuvant radiotherapy in breast cancer is to prevent local recurrence by eradicating residual micro-metastases tissues. Unfortunately, local recurrence and distant metastases are still a problem even in the early stage of breast cancer and with higher incidence in locally advanced breast cancer patients. These recurrences have been documented to occur even after more than 15 years of diagnosis^3^. It is hypothesised that part of the failure in radiotherapy ability to prevent local recurrence in breast cancer is due to the presence of cells that are resistant to irradiation and therefore survive radiotherapy. These cells then repopulated leading to tumour regrowth or recurrent at the previous irradiated area.

The ability of cancer cells to develop acquired radioresistance is associated with multiple factors including signalling pathway dysregulation, activation of DNA damage, existence of cancer stem cells, cancer metabolism changes and epithelial-to-mesenchymal transformation. Hypoxic microenvironments of tumours may also induce cancer cells to acquire aggressive phenotypes that are able to escape treatment^4,5^.

EMT is defined as a biological transition of epithelial cells to mesenchymal cells. This transition involved changes of cell-to-cell interaction and cellular matrix properties leading to enhanced cell migratory and invasiveness^6^. EMT is involved in tumour progression leading to metastasis and development of cancer stem cells. Thus, it has significant and crucial role in cancer cell resistance^7–9^. EMT has been associated with resistance to drug treatment in various studies through the formation of cancer stem cells^10^, enhanced metastases^11^ and activation of survival pathways^12–14^. More evidences showed EMT contributing to radioresistance incidents^14–16^ through modulation of various pathways ^17^.

The association between genes and radioresistance in various types of cancers has been extensively investigated. However, there is an insufficient understanding of the underlying mechanism of radioresistance in EMT due to a lack of model systems^4^. To address this matter, we developed an in vitro model of EMT radioresistance cells (EMT6^RR_MJI^) using epithelial mouse mammary carcinoma cells (EMT6). The differential in gene expressions and pathways involved between EMT6^RR_MJI^ and parental cells were investigated to obtain a more comprehensive profile of the molecular network associated with radioresistance in breast cancer cells. Gene expression and pathway analysis showed increment of expressions in CTLA-4 and PD-1 which led us to further determine the expression of these proteins in EMT6 cell line.

## Results

### Development of acquired EMT6 radioresistance cells (EMT6^RR_MJI^) after fractionated irradiation

**Figure 1:**
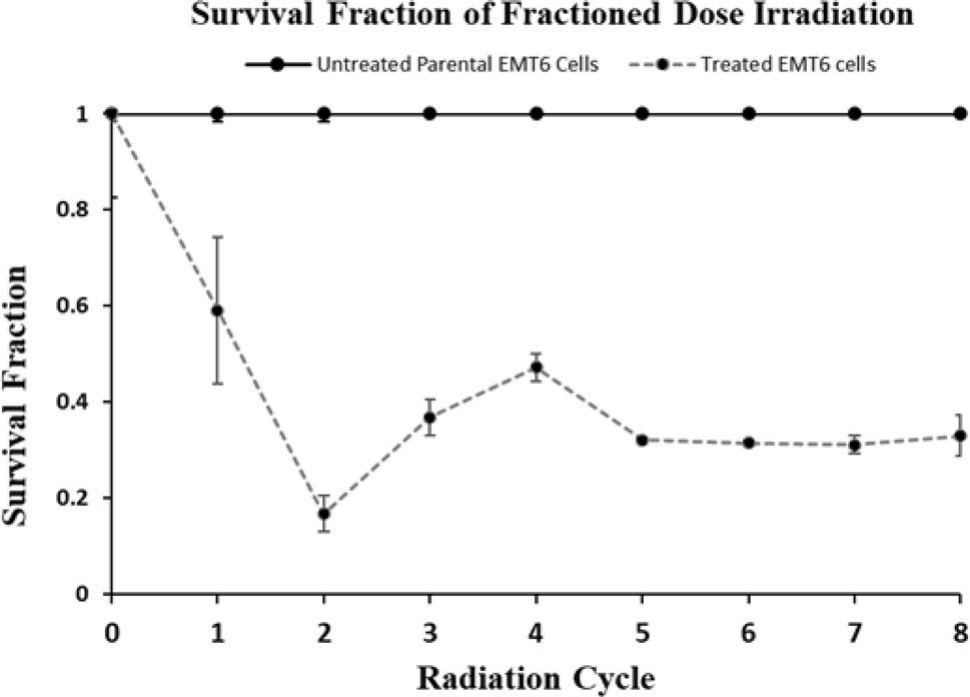
Survival fraction of EMT6 cells after fractionated dose irradiation. The survival fractions of untreated and treated parental EMT6 cells are presented by the respective solid and dotted lines. (Data n = 3, mean ± s.d) (The result presented was partially published^18^).

The survival fraction of parental EMT6 cells after gamma-ray irradiation with a fractionated dose of 2 Gy/cycle up to 16 Gy (8 cycles) was presented in Fig. 1. The mean survival fraction of parental cells was decreased until the 2nd cycle (4 Gy). However, it increased after the 3rd and 4th cycles, but decreased again in the 5th cycle (10 Gy). It plateaued up to the 7th cycle (14 Gy) and slightly increased towards the final 8th cycle (16 Gy). The mean survival fractions were maintained from the 5th to 7th cycles, indicating the EMT6 cells have developed acquired resistance towards gamma-ray. Thus, radioresistance EMT6 cells were successfully developed from parental cells using fractionated irradiation at 2 Gy/cycle. This cell is copyright as EMT6^RR_MJI^ cells with Intellectual Property Corporation of Malaysia (MyIPO) with serial number: CRLY00024431).

### Higher survival fraction between EMT6^RR_MJI^ and parental cells

**Figure 2:**
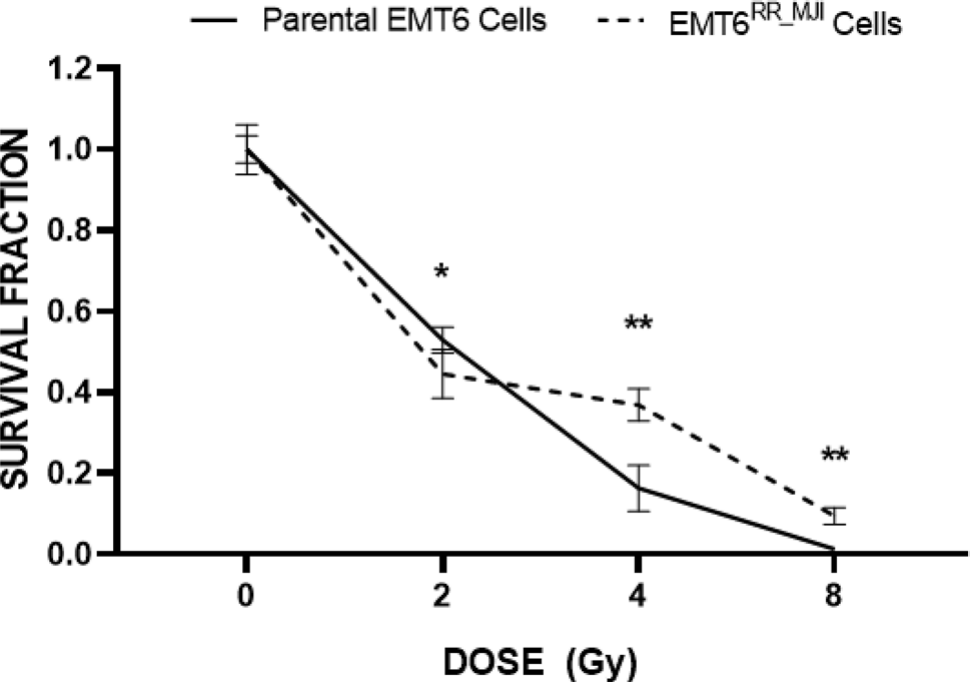
The survival fraction of EMT6^RR_MJI^ and parental cells after being treated with gamma-ray irradiation with 2, 4 and 8 Gy in a single fraction. The survival fractions of EMT6^RR_MJI^ and EMT6 cells are presented by the dotted and solid lines, respectively (*p < 0.05, ***p < 0.0005 significant compared between EMT6^RR_MJI^ with parental cells at the same irradiation dosage) (Data n = 3, mean ± s.d).

The comparison between the survival fraction of EMT6^RR_MJI^ with parental cells was presented in Fig. 2. The mean survival fraction for both cells decreased with increasing doses of irradiation. The survival fraction of EMT6^RR_MJI^ was lower compared to parental cells (p < 0.05) after irradiation with 2 Gy. However, at higher irradiations of 4 Gy and 8 Gy, the survival fractions of EMT6^RR_MJI^ were higher compared to parental cells (p < 0.005), indicating cells have acquired radioresistance to gamma-ray.

### Increased invasion and migration potentials in EMT6^RR_MJI^ compared to parental cells

**Figure 3:**
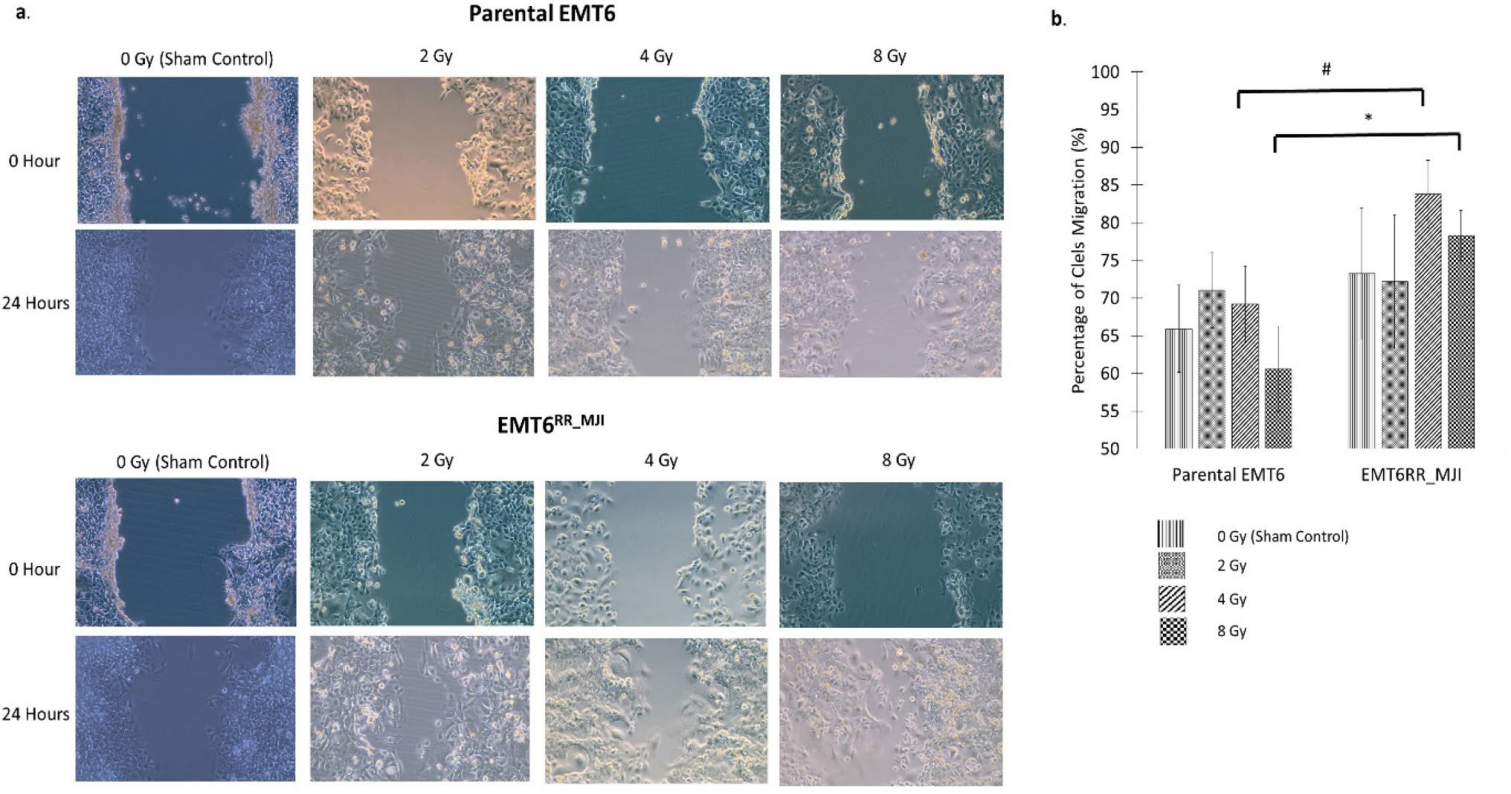
(**a**) Cell migration detected using scratch assay of EMT6^RR_MJI^ and parental cells in treated and sham control groups, 24 h post-irradiation. (**b**) EMT6^RR_MJI^ showed increased migration to 4 Gy and 8 Gy gamma-ray irradiation compared to parental cells after 24 h. (^#^p < 0.01, *p < 0.05) (Data n = 3 ± s.d).

Based on scratch assay, the migration ability of EMT6^RR_MJI^ after irradiation in comparison with parental cells was observed (Fig. 3a,b). Both parental and EMT6^RR_MJI^ cells appeared as the most confluent after irradiation for 36 h causing difficulties in measuring the scratch area for comparison of cell migration between these cells. The migration potential was analysed only at 24 h and a significant increment in EMT6^RR_MJI^ migration was observed compared to parental cells after exposure to 4 Gy and 8 Gy. It was not observed in the control and 2 Gy-treated groups. These findings indicated that the rates of migration and invasion in radioresistance cells increased with an increment in gamma-ray treatment dose.

### Different functional gene pathways regulated in EMT6^RR_MJI^ compared to parental cells

**Figure 4:**
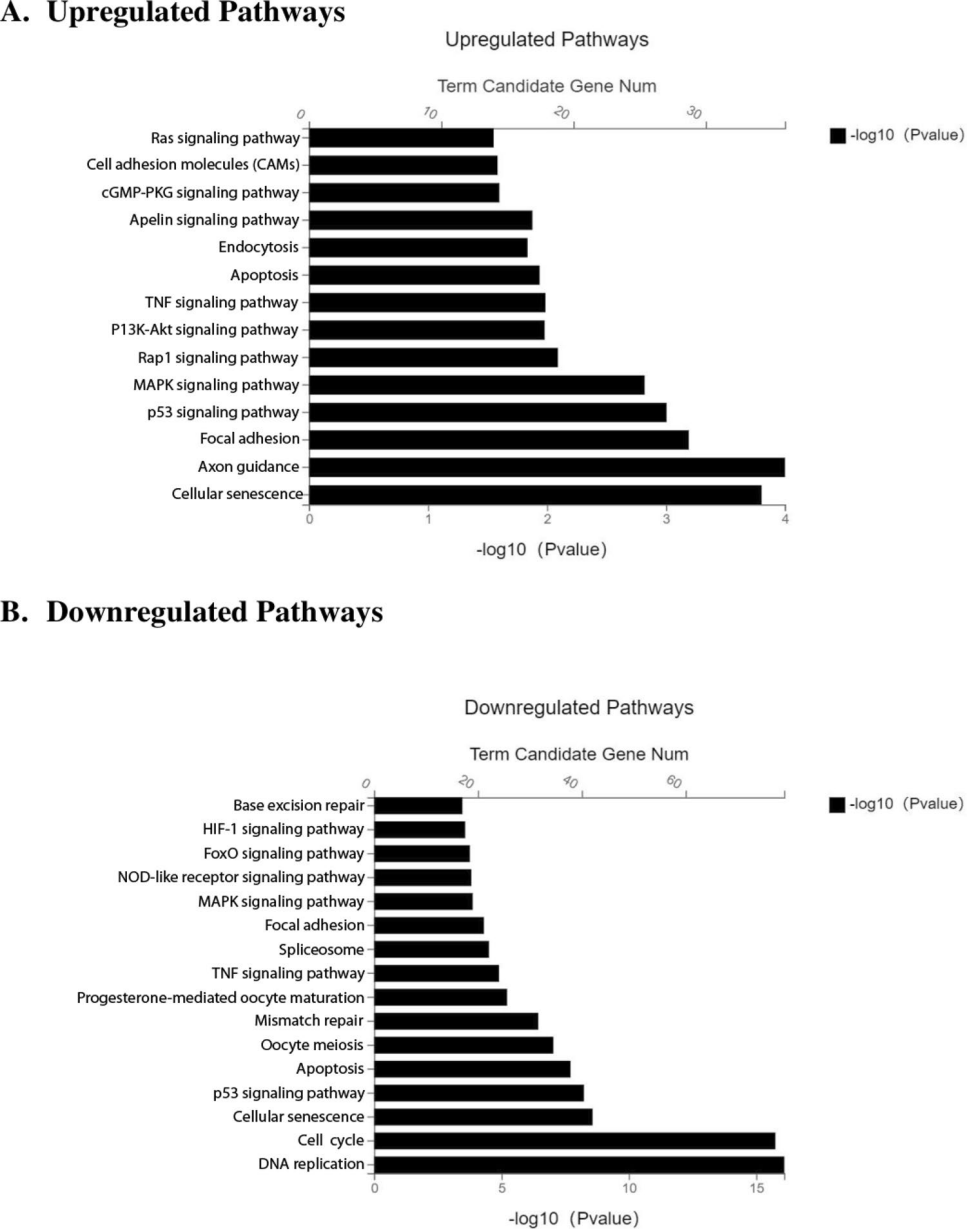
Bioinformatics analysis of the functional pathways contributing to radioresistance in EMT6^RR_MJI^ cells. List of top 10 significant molecular pathways determined by KEGG pathway analysis as described by Kaneisha et al.^19^ for all 418 upregulated genes (**A**) and 430 downregulated genes (**B**) in EMT6^RR_MJI^ cells.

Based on Next Generation Sequencing analysis (NGS), the changes in gene expressions associated pathways were analysed using KEGG pathway classification. A total of 848 genes was identified to exhibit significant differences (p < 0.05) between both parental EMT6 and EMT6^RR_MJI^ cells, with 418 genes upregulated and 430 genes downregulated. The top 10 genes that were either upregulated or downregulated were presented in Fig. 4A,B. In summary, most of the upregulated genes were associated with general oncogenic signalling cascades such as Focal adhesion, Jak-STAT, and PI3K-Akt. While most of the downregulated genes were associated with cellular processes including cell cycle, DNA replication, and p53.

### Potential target genes for acquired radioresistance in EMT6^RR_MJI^ cells

**Figure 5:**
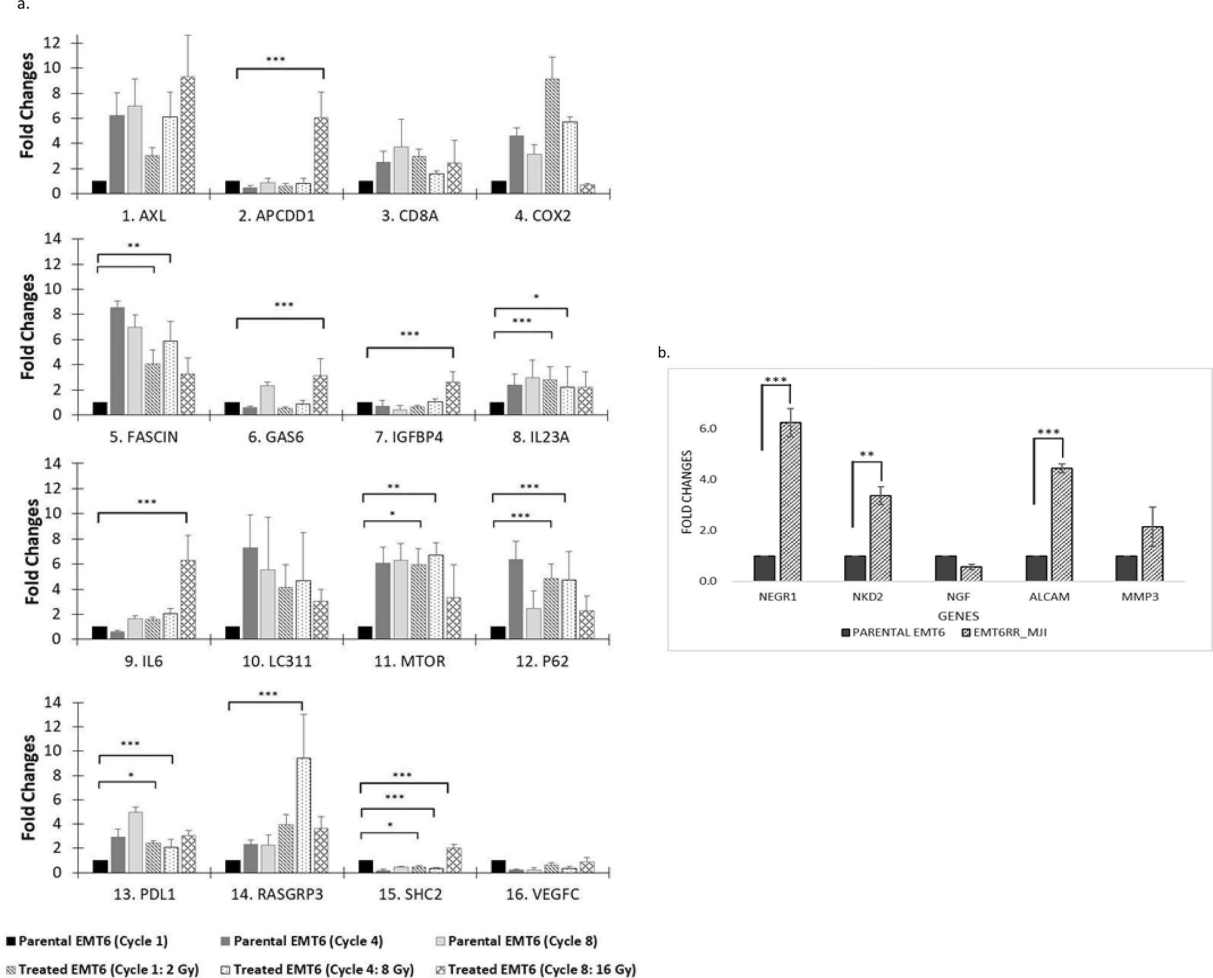
(**a,b**) The expression of target genes associated with acquired radioresistance in EMT6^RR_MJI^ and parental cells was validated by RT-PCR. (**a**) The expression of selected genes that have tenfold changes based on NGS analysis at 1, 4 and 8 cycles between EMT6^RR_MJI^ and parental cells: (**b**) The expression of genes exclusively expressed in EMT6^RR_MJI^ compared to parental cells at 8th cycle; *p < 0.05 vs 0 Gy (1st cycle), **p < 0.01 vs 0 Gy (1st cycle), ***p < 0.001 vs 0 Gy (1st cycle) (Data n = 3, mean ± s.d).

The possible pathway associated with acquiring radioresistance was investigated by analysing the expression of selected genes obtained from NGS data. The expression of these genes was more than tenfold difference in comparison between EMT6^RR_MJI^ with parental cells (16 genes), as well as genes that were exclusively expressed in EMT6^RR_MJI^ cells (5 genes). The expression of these genes was validated using RT-PCR. The selected genes, their functional pathways and Q values were presented in Table 1. The expression of selected genes in all treated groups (EMT6^RR_MJI^ cells) at 1st, 4th and 8th cycles (Cycle 1 = 2 Gy, Cycle 4 = 8 Gy and Cycle 8 = 16 Gy) in comparison to control parental group (EMT6 cells) at similar cycles were presented as fold changes in Fig. 5a.

**Table 1:** List of potential target genes selected based on bioinformatics analysis of functional pathways contributing to radioresistance in EMT6^RR_MJI^ cells.

Genes	Functional signalling pathways	Q-values
IL6	PI3K-AKT, JAK-STAT3, MAPK	0.007
GAS6	PI3K-AKT, JAK-STAT3, EGFR	< 0.001
VEGFC	PI3K-AKT, Focal adhesion	< 0.001
SHC2	PI3K-AKT, RAS/MEK/ERK	< 0.001
AXL	JAK-STAT3, MAPK	0.03
COX-2	EGFR	< 0.001
IL23A	MTOR	0.043
FASCIN	MTOR	0.001
RASGRP3	Tumor immune response	0.001
MTOR	Tumor immune response	0.0072
P62	WNT	0.03
IGFBP4	IGF	< 0.001
LC3II	Autophagy	0.002
PD-L1	JAK-STAT3	0.001
CD8A	JAK-STAT3	0.039
APCDD1	WNT	< 0.001

The expression of APCDD1 (p < 0.001), IL-6 (p < 0.001), SHC2 (p < 0.001), IGFBP4 (p < 0.001), GAS6 (p < 0.01) and AXL (p < 0.001) genes were upregulated at 8th cycle in EMT6^RR_MJI^ compared to EMT6 cells in 1st cycle. The five genes that were exclusively expressed in EMT6^RR_MJI^ cells were also analysed in parental and EMT6-treated groups at 1st and 8th cycles, respectively. The expression of 3 out of 5 genes i.e., NEGR1 (p < 0.001), NKD2 (p < 0.01) and ALCAM (p < 0.001) were significantly higher in EMT6-treated groups compared to parental cells. Even though MMP and NGF were respectively up- and downregulated in EMT6-treated group, these changes were not significant (Fig. 5b). Based on results presented in Fig. 5 and with references to Table 1, the possible pathways responsible for acquired radioresistance in EMT6^RR_MJI^ cells to gamma-ray are concluded as tumour immune response, JAK-STAT3 and PI3K-AKT signalling pathways.

### Protein expressions related to acquired radioresistance in EMT6^RR_MJI^ cells

**Figure 6:**
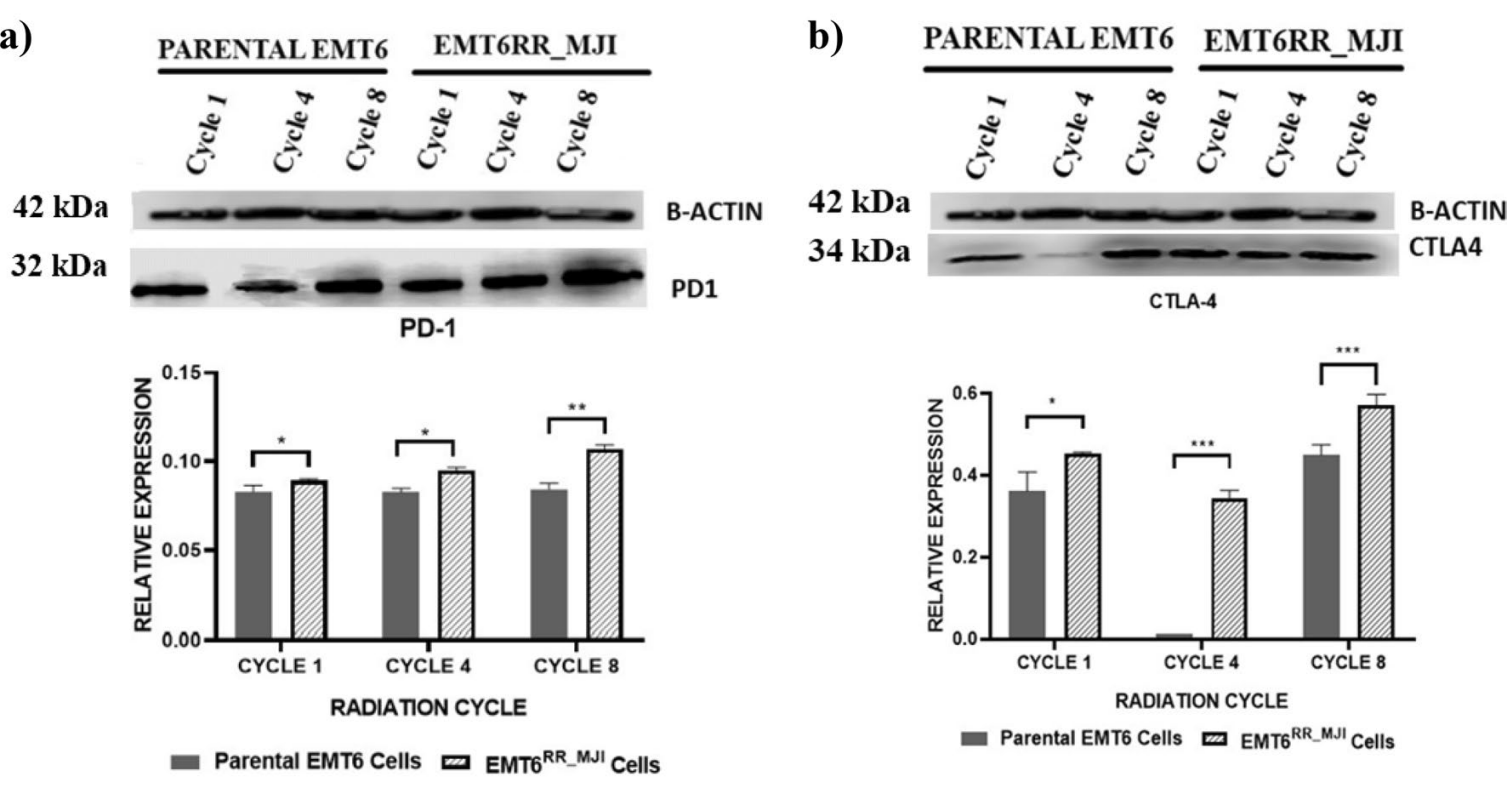
Comparison of PD-1 and CTLA-4 protein expressions between EMT6^RR_MJI^ and parental cells at 1st, 4th and 8th cycles of gamma-ray irradiation. Statistical analysis exhibits an increase of both (**a**) PD-1 and (**b**) CTLA-4 protein expressions in EMT6^RR_MJI^ at all cycles compared to parental cells. The original image blot for beta actin is presented in Supplementary Fig. 2 while for PD-1 and CTLA-4 is presented in Supplementary Fig. 3; *p < 0.05, **p < 0.01, ***p < 0.001 (Data n = 3, mean ± s.d).

Based on gene expression results, the possible involvement of the JAK/STAT/PI3K pathway in the development of EMT6^RR_MJI^ cells was further ascertained by the determination of PD-1 and CTLA-4 expressions, which have been reported to be associated with this pathway. The expression of PD-1 and CTLA-4 proteins was determined in EMT6^RR_MJI^ and parental cells at each cycle. As shown in Fig. 6a, the expression of PD-1 was higher in EMT6^RR_MJI^ compared to parental EMT6 cells at every cycle (Cycle 1, p < 0.05; Cycle 4, p < 0.05; Cycle 8, p < 0.01). Similarly, CTLA-4 protein was also higher in EMT6^RR_MJI^ compared to parental EMT6 cells at every cycle (Cycle 1, p < 0.05; Cycle 4, p < 0.001; Cycle 8, p < 0.001) (Fig. 6b).

## Discussion

The mechanism leading to acquired radioresistance in EMT cells is not well understood due to the lack of a model system available for investigation. Currently, we have successfully developed a novel radioresistance EMT6^RR_MJI^ from parental EMT6 cells to be used as a model system for molecular profiling in relation to cell response caused by irradiation exposure.

EMT6^RR_MJI^ cells was developed using fractionated irradiation by repeatedly exposing parental cells to gamma-ray irradiation at 2 Gy dose for each cycle up to 16 Gy (8th cycles) and allowing cells to recover up to 80% in confluency in between fractions. The general concept of fractionated irradiation is to develop an acquired radioresistance model, i.e., EMT6^RR_MJI^ cells as a mechanistic platform to investigate the underlying mechanism of radioresistance in EMT. The technique used was slightly modified. Previously, prostate cancer, 22Rv1 cells acquired radioresistance and validated by increment in survival fraction after exposure to fractionated irradiation with 2 Gy dose of X-rays once daily for 5 days a week, and continued for a duration of 5 to 7 weeks, with a total accumulative dose of 60 Gy^20–24^. The source of X-ray irradiation was obtained from the RS225 cabinet irradiator (250 keV, 15 Ma) (Gulmay Medical, Surrey, UK)^22^. Similarly, multiple cell lines developed radioresistance within 2 to 3 weeks of fractionated gamma-ray irradiation at 2 Gy for a duration of 5 days and continued for a subsequent 6 weeks in total^24^. The source of the gamma-ray was generated from a Gammacell® 220 Research Irradiator (MDS Nordion, Canada)^25^.

The acquired radioresistance in EMT6^RR_MJI^ cells was validated by irradiating both EMT6^RR_MJI^ and parental cells with gamma-ray, and then subjected to clonogenic and scratch assays. As expected, the survival fraction of EMT6^RR_MJI^ was higher compared to parental cells, thus confirming the radioresistance property of EMT6^RR_MJI^ cells to gamma-ray. The migratory rate of EMT6^RR_MJI^ was also higher compared to parental cells after exposure to gamma-ray at 2 Gy, 4 Gy and 8 Gy. An increment in the migration potential of EMT6^RR_MJI^ cells indicate greater invasive metastatic potential compared to parental cells^24^. These findings concur with the increment in migratory ability of MCF-7 RR and MDA-MB RR radioresistance breast cancer cells compared to their parental cells^4^. Similarly, the expression of mesenchymal markers in doxorubicin-resistant MCF-7 cells increased in correlation with their invasive and metastatic potentials, thus making them resistant to chemotherapy^25^. Hence, this could be a potential cause for the recurrence of cancer and further understanding on how EMT leads to resistance to treatment is needed.

The exploration of potential underlying genes and pathways responsible for rendering EMT cells resistant to irradiation using EMT6^RR_MJI^, was performed by NGS method. IL-6, PDL-1, AXL, GAS6 and APCDD1 genes were upregulated in EMT6^RR_MJI^ after gamma-ray irradiation at 8th cycle compared to parental cells. Based on the association between immune system and inflammatory processes within tumours, it is suggested that inflammation may provide tumour-promoting signals contributing to poorer outcomes^26^. One of the genes suggested was interleukin (IL)-6. This gene and its family members were identified as key players and biomarkers in the pathogenesis of several chronic inflammatory diseases including cancer through the initiation of tumour, subsequent growth and metastasis^27–30^. Hyperactivity of IL-6 through JAK/STAT3 pathway leads to an increase in tumour proliferation, invasiveness, metastatic ability and chemoresistance in colorectal cancer through enhancement of EMT; while actively suppressing antitumour immune response through downregulation of programmed death-ligand 1 (PD-L1)^31^. The upregulation of IL-6 is in accordance with other studies, indicating its contribution to radioresistance in EMT cells. Its upregulation in tumour microenvironment was associated with malignant phenotypes and subsequent resistance to chemotherapy^21,26,30^. The expression levels of IL-6 and its family members can be useful to diagnose prognosis of relapse-free survival and recurrence in cancer patients^21,30,32^.

As a member of an immunoglobulin superfamily, PD-1 is one of the most important inhibitory checkpoint molecules that governed T cell activities. PD-1 was expressed at low levels in immune system resting cells^33^. Overexpression of PD-1 enabled tumour cells to be protected from cytotoxic T lymphocytes^34^ and the development of anti-PD-1/PD-L1 antibodies is believed to be the core area in cancer immunotherapy^35,36^. Overexpression of PD-L1 predicted the clinical outcomes of patients with lower-grade glioma after receiving radiotherapy^37^. Mice lacking in PD-1 developed autoimmune disorders, indicating its role in the regulation of immune response. Upregulation of PD-L1 was consistent with immunohistochemistry results of patients with oesophageal squamous cell carcinoma (ESCC) after irradiation with X-rays. The investigation of both proteins in resistance human breast cancer model that can mimic the tumour microenvironment architecture of breast cancer will increase the significance of the data. The availability of a feline model for breast cancer microenvironment study enables scientists to determine the involvement of PD-1 and PD-L1 proteins in the radioresistance of felines^38,39^.

PD-1 mRNA was upregulated in 20% of breast tumours as compared to normal breast tissue^40,41^. Another study reported overexpression of PD-1/PD-L1 mRNA among half (55% to 59%) of breast cancer patients^40,42^. Based on the invasive lobular carcinoma histologic sub-type, breast cancers were divided into immune- and hormone-related, with the former cases expressing higher mRNA expression of PD-L1, PD-1, and CTLA-4 ^43^. PD-L1 triggered an inhibitory signalling pathway preventing T-cell activation^32^. By this way, immune-mediated cell death was avoided and continued to proliferate and survived in the tumour microenvironment^44^ thus escaping from treatment and causing resistance. PD-L1 was expressed on the cell surface of several types of cancers including breast cancer^45,46^.

PD-1 and its ligand (PD-L1), as well as CTLA-4 immune checkpoint pathways reduce T-cells activation to preserve peripheral tolerance. Cancer cells were able to utilize these pathways to generate an immunosuppressive state that allowed tumours to grow and proliferate rather than destroyed by immune system^47^. CTLA-4 and PD-1 proteins were overexpressed in both immune T-cells and various cancers such as melanoma^48,49^, ovarian, breast, prostate, and pancreatic^50,51^. Accordingly, these proteins were expressed in EMT6^RR_MJI^ cells. Blocking T cell inhibitory pathways has become the new cancer treatment approach and immunological suppressive pathways involving CTLA-4 and PD-1 are actively studied^52,53^. The severity of patients diagnosed with melanoma, lung, renal, and bladder cancers significantly improved and benefited from anti-CTLA-4 and anti-PD-1 monotherapies^54–58^. A reduction in tumour development obtained by blocking CTLA-4 or PD-1 pathways, provides justification for the inhibition of immune checkpoints in cancer treatment^59,60^.

CTLA-4 acted as a cell surface receptor and inhibited T cells from transmitting immunological signals^61^. CTLA-4 and Tregs (regulatory T cells) signify complementary and overlapping mechanisms of immune tolerance. Tregs’ suppressive activity was decreased due to impairment of CTLA-4 ^62,63^. CTLA-4 competes with its homologue, CD28 for ligand CD80/CD86, thus interfering with CD28-mediated T cell activation and decreasing response^64^. Overexpression of CTLA-4 protein in four different breast cancer and tumorigenic cell lines indicates its association with resistance towards radiotherapy^65^. Accordingly, CTLA-4 overexpression was associated with poor survival in breast cancer patients^55,65^. The association between a better prognosis with CTLA-4 overexpression in tumour infiltrating lymphocytes (TILs) implied the crucial role of CTLA-4 in escape immune response by tumours^40,66^.

Apart from IL-6 and PD-1, three other genes i.e., AXL, GAS6 and APCDD1 were also overexpressed in EMT6^RR_MJI^ cells, indicating an association with acquiring radioresistance to gamma-ray. AXL is a tyrosine kinase receptor expressed in various cancer types and associated with poor prognosis and resistance to treatment^44^. GAS6/AXL was involved in promoting tumour cells’ proliferation, survival, and angiogenesis. AXL was identified as an essential migration regulator and inducer of EMT^44,67^. Its overexpression protects breast cancer cells from apoptosis, hence escaping treatment and subsequently resistance to radiotherapy. APCDD1 is an inhibitor of WNT signalling pathway^68^ and a promoter for colorectal cancer proliferation^31^ and tumorigenesis^44^. However, APCDD1 expression was decreased in invasive breast cancer cells compared to non-invasive cells^69^, due to its inhibitory function in WNT signalling pathway. Despite numerous studies, there is very little scientific understanding regarding the association of APCDD1 with radioresistance.

In conclusion, radioresistance cells, EMT6^RR_MJI^ was successfully developed from parental cells after 8 cycles of radiation (2 Gy each cycle). Using NGS and bioinformatic software, several overexpressed genes were identified and believed to be associated with the development of acquired radioresistance in EMT6^RR_MJI^ through JAK/STAT/PI3K pathway. PD-1 and CTLA-4, that were associated with JAK/STAT/PI3K pathway was later confirmed overexpressed in EMT6^RR_MJI^ by Western blot. Further study is required to develop an in vivo model mimicking tumour microenvironment architecture of breast cancer to clarify comprehensive pathways as target for immunological suppression and subsequently reducing tumour development.

## Methods

### Cells and culture condition

EMT6 mouse mammary cancer cells or EMT6 parental cells (American Type Culture Collection, Virginia, USA) were cultured in T25 flask containing 12 mL Dulbecco’s modified eagle’s medium (DMEM), supplemented with 10% foetal bovine serum (FBS) and 1% streptomycin and penicillin up to the 4th passages. Cells were incubated in a humidified atmosphere with 5% CO_2_ at 37 °C until 70% to 80% confluence before irradiated with gamma-ray. The culture media components used were tissue culture grade (Thermo Fisher Scientific, USA).

### Development of EMT6^RR_MJI^ through irradiation of parental cells

Parental EMT6 cells cultured in T25 flasks (ThermoFisher Scientific, USA) containing 12 mL complete medium were exposed to gamma-ray irradiation using a Gamma cell 220 Excel (MDS NORDION/GC 220 E, Department of Nuclear Science, Faculty of Science and Technology, Universiti Kebangsaan Malaysia) with an initial dose of 2 Gy; and then repeated with another 2 Gy for each cycle to mimic the irradiation protocol in human until radioresistance cells were developed. The development of radioresistance in these cells was measured using survival fraction data obtained from clonogenic assay.

After the initial dose of irradiation (2 Gy), cells were incubated again in 5% CO_2_ for 2 h before harvested and divided into two parts. The first half was collected and adjusted to specific seeding densities for clonogenic assay to determine the survival fraction. The other half (3 × 10^5^ cells) was grown in T25 flask until 70 to 80% confluency and used for the next cycle of irradiation. For each irradiation cycle, cells were cultured in T25 flask in triplicate. During each cycle of irradiation, survival fraction was measured. The radioresistance cells were successfully developed when survival fraction was higher, and constant compared to previous cycle and the protocol was ceased (Supplementary Fig. 1).

### Clonogenic assay

Clonogenic assay is a gold standard to measure radiation effect by measuring survival fraction of irradiated cells ^70,71^. Briefly, all groups of irradiated (treated EMT6 cells) and sham control (parental EMT6 cells) were incubated at 37 °C for 2 h, trypsinized and diluted to the desired concentrations for clonogenic plating in separate 100 mm petri dishes. Cells were allowed to grow and form colonies within eight days^72^. A colony is defined to consist of at least 50 cells. Colonies fixation and staining were conducted by staining cells with 0.5% crystal violet in 50/50 methanol/water for 5 min. Cells were rinsed with tap water and left to dry at room temperature. Cell colonies were scanned using a scanner (Epson V600, Japan) and counted using ImageJ software version 1.53 k (https://imagej.nih.gov/ij/index.html). The plating efficiency of sham control and all treatment groups to gamma-ray irradiation was calculated using the formula:

$$\left( {{\text{Number of colonies}}/{\text{numbers of cell-seeded}}} \right) \, \times { 1}00.$$

The survival fraction of cells for each treatment was calculated by normalizing the plating efficiency of treatment with sham control group.

### Confirmation of radioresistance EMT6^RR_MJI^ cells development

#### Irradiation with different doses of gamma-ray

The development of EMT6^RR_MJI^ cells from their parental cells was confirmed by further irradiating sham control (parental EMT6 cells) and radioresistance EMT6 cell groups with different dosages of gamma-ray at 2 Gy, 4 Gy and 8 Gy. Both cells’ groups have similar passage numbers. The survival fraction of both cell groups was determined using clonogenic assay.

#### Scratch assay

A total of 2.5 × 10^5^ parental and EMT6^RR_MJI^ cells were plated separately into each well of 12-well plates for 48 h up to 80% confluency. Cells were wounded by scratching with a 10 µL pipette tip. The debris was later removed, and cells were washed once with 1 mL growth medium. This is to assure smooth edges of scratch area. Cells were then incubated in DMEM containing 0.5% FBS. Cell migration was assessed by a monolayer gap closure of scratch area. Here, the image of scratch area was captured at initial time (0 h) and after 24-, 36- and 72-h using an inverted microscope (Leica, Germany). Then, the area was measured by ImageJ software version 1.53 k (https://imagej.nih.gov/ij/index.html). software using the wound healing size tool plugin^73^. Data were calculated using the calculation below and presented as a percentage of cell migration.

$${\text{Percentage of migration}}: \, \left( {{1}00 \, {-}{\text{ A}}_{{2}} } \right) \, {-} \, \left( {{1}00 \, {-}{\text{ A}}_{{1}} } \right)/\left( {{1}00 \, {-}{\text{ A}}_{{1}} } \right),$$

where A_1_ and A_2_ are the respective scratch areas at initial time and after 24 h.

### Comparison of gene analysis between EMT6^RR_MJI^ with parental cells

#### RNA extraction and next generation sequencing

Total RNA was extracted from both cells (2.5 × 10^5^) using a Macherey–Nagel RNA extraction kit (MN, Germany) following the manufacturer’s instructions. A NanoDropTM Spectrophotometer (ND-1000 Thermo Fisher Scientific, USA) was used to quantify RNA and assess contamination. NGS was generated using RNA-seq technology. Sequencing was carried out using BGISEQ-500 platform at BGI Genomics Co., Ltd (BGI Co., Ltd, Singapore). The mRNA was enriched with oligo(dT)-attached magnetic beads. After purification, mRNA was cut into smaller fragments, and reverse transcription of cDNA was initiated by a random N6 primer. A-tailing Mix and RNA Index Adapters were ligated to the ends of cDNA fragments. Ligation products were then purified and amplified by PCR and validated for quality control. Reads were generated on the BGIseq500 platform (BGI) after the heating denaturation of PCR products. RNA-seq reads were mapped to the reference genome^74^. Gene expression levels were quantified using RSEM software^75^. The changes in gene expressions were determined by comparing treatments (radioresistance EMT6 cells) with control (parental EMT6 cells) groups. Genes of interest were selected based on the expression of more than 10 for further validation using RT-PCR. The differential molecular pathways regulated by EMT6^RR_MJI^ cells versus parental EMT6 cells were analysed and identified by KEGG Pathway Classification analysis^19^.

#### Gene expression analysis of shortlisted genes using RT-PCR

Based on NGS results, 21 genes were identified with fold changes at cut-off of ten (16 expressed in both parental EMT6 and EMT6^RR_MJI^, while 5 genes expressed only in EMT6^RR_MJI^ cells). Primer sequences for all genes were presented in Supplementary Table S1. One-step RT-PCR method (Bioline SensiFAST™ SYBR® NO-ROX kit) was used according to the manufacturer’s protocol (Bioline, UK). The reaction mix composition was listed in Supplementary Table S2. Genes detected by Biorad CFX96 instrument (Bio-rad, USA) and PCR cycle conditions were listed in Supplementary Table S3. Gene expression was analysed as fold change using DeltaDeltaCT to the housekeeping gene.

### Comparison of protein analysis between EMT6^RR_MJI^ with parental cells

Total protein was extracted from both cells using radioimmunoprecipitation assay (RIPA) lysis buffer (Abcam, UK). Protein concentrations were determined using a Pierce BCA Protein Assay Kit (Thermo Fisher Scientific, USA). An equal amount of protein from both cells was separated using 12% SDS–polyacrylamide gel and transferred onto a nitrocellulose membrane. The blotted membranes were blocked with Tris-buffered saline containing 5% non-fat milk to avoid nonspecific binding sites before the addition of primary antibodies i.e., anti-PD1 (EPR20665, 1:1000, Abcam, UK), anti-CTLA4 (CAL49, 1:1000, Abcam, UK), and anti-beta-actin (EPR21241, 1:5000, Abcam, UK). Beta-actin served as an internal control. After 24 h of incubation at 4 °C, the membrane was incubated again for 1 h with secondary antibody i.e., Immunoglobulin G (IgG) (1:100,000, Abcam, UK) at room temperature. Finally, the proteins were detected using Enhanced Chemiluminescence (ECL) Substrate Kit (Abcam, UK).

### Statistical analysis

Each experiment was carried out in triplicates and data was expressed as mean ± standard deviation. Unpaired *t*-test and one-way ANOVA with Bonferroni multiple comparison test were used to analyse data obtained from clonogenic and RT PCR assays, respectively. Significance was set at p < 0.05. Statistical analysis was performed using IBM SPSS Statistics for Windows version 23 (IBM Corp. in Armonk, NY) and graphs were generated with GraphPad Prism version 8.0 for Windows (San Diego, California USA).

## Supplementary Information

Supplementary Figure 1.

Supplementary Figure 2.

Supplementary Figure 3.

Supplementary Table S1.

Supplementary Table S2.

Supplementary Table S3.

## Data Availability

The datasets generated in the current study are available in the National Centre for Biotechnology Information (NCBI) repository database with accession number of PRJNA881849 (https://www.ncbi.nlm.nih.gov/search/all/?term=PRJNA881849).

## Abbreviations

APCDD1: Adenomatosis polyposis downregulated 1
AXL: AXL receptor tyrosine kinase
CTLA-4: Cytotoxic-lymphocyte antigen-4
DMEM: Dulbecco’s modified eagle’s medium
ECL: Enhanced chemiluminescence
EMT: Epithelial-mesenchymal transition
FBS: Foetal bovine serum
GAS6: Growth-arrest-specific gene 6
IgG: Immunoglobulin G
IL-6: Interleukin-6
NGS: Next generation sequencing
PD-1: Programmed death-1
PD-L1: Programmed death-ligand 1
RIPA: Radioimmunoprecipitation assay
TILs: Tumour infiltrating lymphocytes
WNT: Wnt family member

## References

[1] Sung H, Ferlay J, Siegel RL (2021). Global cancer statistics 2020: GLOBOCAN estimates of incidence and mortality worldwide for 36 cancers in 185 countries. CA Cancer J. Clin..

[2] Cardoso F, Kyriakides S, Ohno S (2019). Early breast cancer: ESMO clinical practice guidelines for diagnosis, treatment and follow-up. Ann. Oncol..

[3] Spronk I, Schellevis FG, Burgers JS, de Bock GH, Korevaar JC (2018). Incidence of isolated local breast cancer recurrence and contralateral breast cancer: A systematic review. Breast.

[4] Gray M, Turnbull AK, Meehan J (2020). Comparative analysis of the development of acquired radioresistance in canine and human mammary cancer cell lines. Front. Vet. Sci..

[5] Barker HE, Paget JTE, Khan AA, Harrington KJ (2015). The tumour microenvironment after radiotherapy: Mechanisms of resistance and recurrence. Nat. Rev. Cancer.

[6] Roche J (2018). The epithelial-to-mesenchymal transition in cancer. Cancers (Basel).

[7] Lambert AW, Pattabiraman DR, Weinberg RA (2017). Emerging biological principles of metastasis. Cell.

[8] Moustakas A, de Herreros AG (2017). Epithelial-mesenchymal transition in cancer. Mol. Oncol..

[9] Acloque H, Adams MS, Fishwick K, Bronner-Fraser M, Nieto MA (2009). Epithelial-mesenchymal transitions: The importance of changing cell state in development and disease. J. Clin. Investig..

[10] Singh A, Settleman J (2010). EMT, cancer stem cells and drug resistance: An emerging axis of evil in the war on cancer. Oncogene.

[11] Gooding AJ, Schiemann WP (2020). Epithelial–mesenchymal transition programs and cancer stem cell phenotypes: Mediators of breast cancer therapy resistance. Mol. Cancer Res..

[12] Wilson C, Ye X, Pham T (2014). AXL inhibition sensitizes mesenchymal cancer cells to antimitotic drugs. Cancer Res..

[13] Wilson C, Nicholes K, Bustos D (2014). Overcoming EMT-associated resistance to anti-cancer drugs via Src/FAK pathway inhibition. Oncotarget.

[14] Jiang YH, You KY, Bi ZF, Li LT, Mo HQ, Liu YM (2018). The relationship between the radioresistance of pancreatic cancer cell SW1990 and the induction of the epithelial-mesenchymal transition: An in vitro study. Zhonghua Yi Xue Za Zhi.

[15] Nantajit D, Lin D, Li JJ (2015). The network of epithelial-mesenchymal transition: Potential new targets for tumor resistance. J. Cancer Res. Clin. Oncol..

[16] Theys J, Jutten B, Habets R (2011). E-Cadherin loss associated with EMT promotes radioresistance in human tumor cells. Radiother. Oncol..

[17] Zhou S, Zhang M, Zhou C, Wang W, Yang H, Ye W (2020). The role of epithelial-mesenchymal transition in regulating radioresistance. Crit. Rev. Oncol./Hematol..

[18] Sham NFR, Hasan N, Hasani NAH, Karim MK, Ibahim MJ (2020). Study of morphological changes and survival fraction in EMT6 cell line post-gamma ray irradiation. J. Phys. Conf. Ser..

[19] Kanehisa M, Araki M, Goto S (2008). KEGG for linking genomes to life and the environment. Nucleic Acids Res..

[20] Gray M, Turnbull AK, Ward C (2019). Development and characterisation of acquired radioresistant breast cancer cell lines. Radiat. Oncol..

[21] Kuwahara Y, Li L, Baba T (2009). Clinically relevant radioresistant cells efficiently repair DNA double-strand breaks induced by X-rays. Cancer Sci..

[22] McDermott N, Meunier A, Mooney B (2016). Fractionated radiation exposure amplifies the radioresistant nature of prostate cancer cells. Sci. Rep..

[23] Nguyen AM, Zhou J, Sicairos B, Sonney S, Du Y (2020). Upregulation of CD73 confers acquired radioresistance and is required for maintaining irradiation-selected pancreatic cancer cells in a mesenchymal state. Mol. Cell Proteom..

[24] van den Berg J, Castricum KCM, Meel MH (2020). Development of transient radioresistance during fractionated irradiation in vitro. Radiother. Oncol..

[25] Wang C, Jin H, Wang N (2016). Gas6/Axl axis contributes to chemoresistance and metastasis in breast cancer through Akt/GSK-3β/β-catenin signaling. Theranostics.

[26] Fisher DT, Appenheimer MM, Evans SS (2014). The two faces of IL-6 in the tumor microenvironment. Semin. Immunol..

[27] Mantovani A, Allavena P, Sica A, Balkwill F (2008). Cancer-related inflammation. Nature.

[28] Naugler WE, Karin M (2008). The wolf in sheep’s clothing: The role of interleukin-6 in immunity, inflammation and cancer. Trends Mol. Med..

[29] Rose-John S (2012). IL-6 Trans-signaling via the soluble IL-6 receptor: Importance for the pro-inflammatory activities of IL-6. Int. J. Biol. Sci..

[30] Guo Y, Xu F, Lu T, Duan Z, Zhang Z (2012). Interleukin-6 signaling pathway in targeted therapy for cancer. Cancer Treat Rev..

[31] Takahashi M, Fujita M, Furukawa Y (2002). Isolation of a novel human gene, APCDD1, as a direct target of the β-catenin/T-cell factor 4 complex with probable involvement in colorectal carcinogenesis. Cancer Res..

[32] Chen MF, Chen PT, Chen WC, Lu MS, Lin PY, Lee KD (2016). The role of PD-L1 in the radiation response and prognosis for esophageal squamous cell carcinoma related to IL-6 and T-cell immunosuppression. Oncotarget.

[33] Keir ME, Francisco LM, Sharpe AH (2007). PD-1 and its ligands in T-cell immunity. Curr. Opin. Immunol..

[34] Zou W, Chen L (2008). Inhibitory B7-family molecules in the tumour microenvironment. Nat. Rev. Immunol..

[35] Nishimura H, Okazaki T, Tanaka Y (2001). Autoimmune dilated cardiomyopathy in PD-1 receptor-deficient mice. Science.

[36] Nishimura H, Nose M, Hiai H, Minato N, Honjo T (1999). Development of lupus-like autoimmune diseases by disruption of the PD-1 gene encoding an ITIM motif-carrying immunoreceptor. Immunity.

[37] Jang BS, Kim IA (2018). A radiosensitivity gene signature and PD-L1 predict the clinical outcomes of patients with lower grade glioma in TCGA. Radiother. Oncol..

[38] Nascimento C, Urbano AC, Gameiro A, Ferreira J, Correia J, Ferreira F (2020). Serum PD-1/PD-L1 levels, tumor expression and PD-L1 somatic mutations in HER2-positive and triple negative normal-like feline mammary carcinoma subtypes. Cancers (Basel).

[39] Nascimento C, Ferreira F (2021). Tumor microenvironment of human breast cancer, and feline mammary carcinoma as a potential study model. Biochim. Biophys. Acta Rev. Cancer.

[40] Voutsadakis IA (2016). Immune blockade inhibition in breast cancer. Anticancer Res..

[41] Sabatier R, Finetti P, Mamessier E (2015). Prognostic and predictive value of PDL1 expression in breast cancer. Oncotarget.

[42] Schalper KA, Velcheti V, Carvajal D (2014). In situ tumor PD-L1 mRNA expression is associated with increased TILs and better outcome in breast carcinomas. Clin. Cancer Res..

[43] Michaut M, Chin SF, Majewski I (2016). Integration of genomic, transcriptomic and proteomic data identifies two biologically distinct subtypes of invasive lobular breast cancer. Sci. Rep..

[44] Tanaka M, Siemann DW (2020). Gas6/Axl signaling pathway in the tumor immune microenvironment. Cancers (Basel).

[45] Francisco LM, Salinas VH, Brown KE (2009). PD-L1 regulates the development, maintenance, and function of induced regulatory T cells. J. Exp. Med..

[46] Keir ME, Butte MJ, Freeman GJ, Sharpe AH (2008). PD-1 and its ligands in tolerance and immunity. Annu. Rev. Immunol..

[47] Buchbinder EI, Desai A (2016). CTLA-4 and PD-1 pathways: Similarities, differences, and implications of their inhibition. Am. J. Clin. Oncol..

[48] Ehlerding EB, England CG, Majewski RL (2017). ImmunoPET imaging of CTLA-4 expression in mouse models of non-small cell lung cancer. Mol. Pharm..

[49] Laurent S, Queirolo P, Boero S (2013). The engagement of CTLA-4 on primary melanoma cell lines induces antibody-dependent cellular cytotoxicity and TNF-alpha production. J. Transl. Med..

[50] Grenga I, Donahue RN, Lepone L, Bame J, Schlom J, Farsaci B (2014). PD-L1 and MHC-I expression in 19 human tumor cell lines and modulation by interferon-gamma treatment. J. Immunother. Cancer.

[51] Zheng Y, Fang YC, Li J (2019). PD-L1 expression levels on tumor cells affect their immunosuppressive activity. Oncol. Lett..

[52] Sharma P, Allison JP (2015). The future of immune checkpoint therapy. Science..

[53] Sharma P, Allison JP (2015). Immune checkpoint targeting in cancer therapy: Toward combination strategies with curative potential. Cell.

[54] Borghaei H, Paz-Ares L, Horn L (2015). Nivolumab versus docetaxel in advanced nonsquamous non-small-cell lung cancer. N. Engl. J. Med..

[55] Hodi FS, O’Day SJ, McDermott DF (2010). Improved survival with ipilimumab in patients with metastatic melanoma. N. Engl. J. Med..

[56] Rosenberg JE, Hoffman-Censits J, Powles T (2016). Atezolizumab in patients with locally advanced and metastatic urothelial carcinoma who have progressed following treatment with platinum-based chemotherapy: A single-arm, multicentre, phase 2 trial. The Lancet.

[57] Gao J, Shi LZ, Zhao H (2016). Loss of IFN-γ pathway genes in tumor cells as a mechanism of resistance to anti-CTLA-4 therapy. Cell.

[58] Motzer RJ, Escudier B, McDermott DF (2015). Nivolumab versus everolimus in advanced renal cell carcinoma. N. Engl. J. Med..

[59] Leach DR, Krummel MF, Allison JP (1996). Enhancement of antitumor immunity by CTLA-4 blockade. Science.

[60] Son CH, Bae J, Lee HR, Yang K, Park YS (2017). Enhancement of antitumor immunity by combination of anti-CTLA-4 antibody and radioimmunotherapy through the suppression of Tregs. Oncol. Lett..

[61] Alberts, B., Johnson, A., Lewis, J., Raff, M., Roberts, K. & Walter, P. Helper T cells and lymphocyte activation. *Molecular Biology of the Cell 4th Edition*. https://www.ncbi.nlm.nih.gov/books/NBK26827/ (Accessed 5 January 2022) (2002).

[62] Takahashi T, Tagami T, Yamazaki S (2000). Immunologic self-tolerance maintained by CD25(+)CD4(+) regulatory T cells constitutively expressing cytotoxic T lymphocyte-associated antigen 4. J. Exp. Med..

[63] Wing K, Onishi Y, Prieto-Martin P (2008). CTLA-4 control over Foxp3+ regulatory T cell function. Science.

[64] Rudd CE, Taylor A, Schneider H (2009). CD28 and CTLA-4 coreceptor expression and signal transduction. Immunol. Rev..

[65] Denkert C, von Minckwitz G, Brase JC (2015). Tumor-infiltrating lymphocytes and response to neoadjuvant chemotherapy with or without carboplatin in human epidermal growth factor receptor 2-positive and triple-negative primary breast cancers. J. Clin. Oncol..

[66] Barrueto L, Caminero F, Cash L, Makris C, Lamichhane P, Deshmukh RR (2020). Resistance to checkpoint inhibition in cancer immunotherapy. Transl. Oncol..

[67] Vuoriluoto K, Haugen H, Kiviluoto S (2011). Vimentin regulates EMT induction by slug and oncogenic H-Ras and migration by governing Axl expression in breast cancer. Oncogene.

[68] Cruciat CM, Niehrs C (2013). Secreted and transmembrane wnt inhibitors and activators. Cold Spring Harb. Perspect. Biol..

[69] Cho SG (2017). APC downregulated 1 inhibits breast cancer cell invasion by inhibiting the canonical WNT signaling pathway. Oncol. Lett..

[70] Puck TT, Marcus PI (1956). Action of X-rays on mammalian cells. J. Exp. Med..

[71] Matsui T, Nuryadi E, Komatsu S (2019). Robustness of clonogenic assays as a biomarker for cancer cell radiosensitivity. Int. J. Mol. Sci..

[72] Ibahim MJ, Yang Y, Crosbie JC (2016). Eosinophil-associated gene pathways but not eosinophil numbers are differentially regulated between synchrotron microbeam radiation treatment and synchrotron broad-beam treatment by 48 hours postirradiation. Radiat. Res..

[73] Suarez-Arnedo A, TorresFigueroa F, Clavijo C, Arbeláez P, Cruz JC, Muñoz-Camargo C (2020). An image J plugin for the high throughput image analysis of in vitro scratch wound healing assays. PLoS ONE.

[74] Langmead B, Trapnell C, Pop M, Salzberg SL (2009). Ultrafast and memory-efficient alignment of short DNA sequences to the human genome. Genome Biol..

[75] Li B, Dewey CN (2011). RSEM: Accurate transcript quantification from RNA-Seq data with or without a reference genome. BMC Bioinform..

