# Supplementary material for: Acquired radioresistance in EMT6 mouse mammary carcinoma cell line is mediated by CTLA-4 and PD-1 through JAK/STAT/PI3K pathway
Source: Sci Rep. 2023 Feb 22;13:3108. doi: 10.1038/s41598-023-29925-x (PMC9946948; doi:10.1038/s41598-023-29925-x)

Supplementary Figure 1: The flowchart of study design to produce radioresistance cell until 8th cycle of radiation.

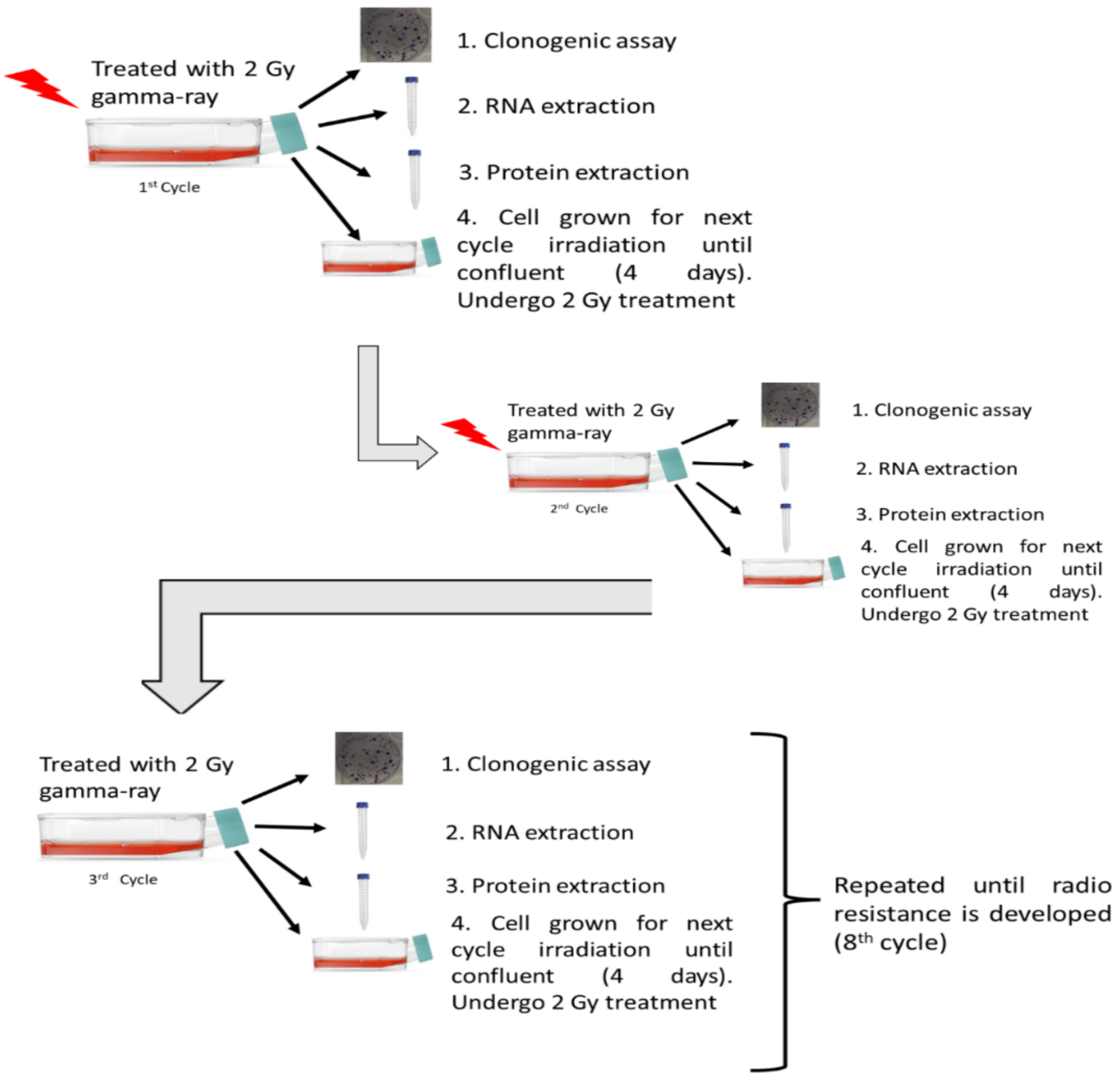

Supplement: Supplementary file 1 — Supplementary Figure 1. [file 41598_2023_29925_MOESM1_ESM.pdf]
