# Supplementary figures and images for: Acquired radioresistance in EMT6 mouse mammary carcinoma cell line is mediated by CTLA-4 and PD-1 through JAK/STAT/PI3K pathway
Source: Sci Rep. 2023 Feb 22;13:3108. doi: 10.1038/s41598-023-29925-x (PMC9946948; doi:10.1038/s41598-023-29925-x)

## B-ACTIN BAND PROTEIN

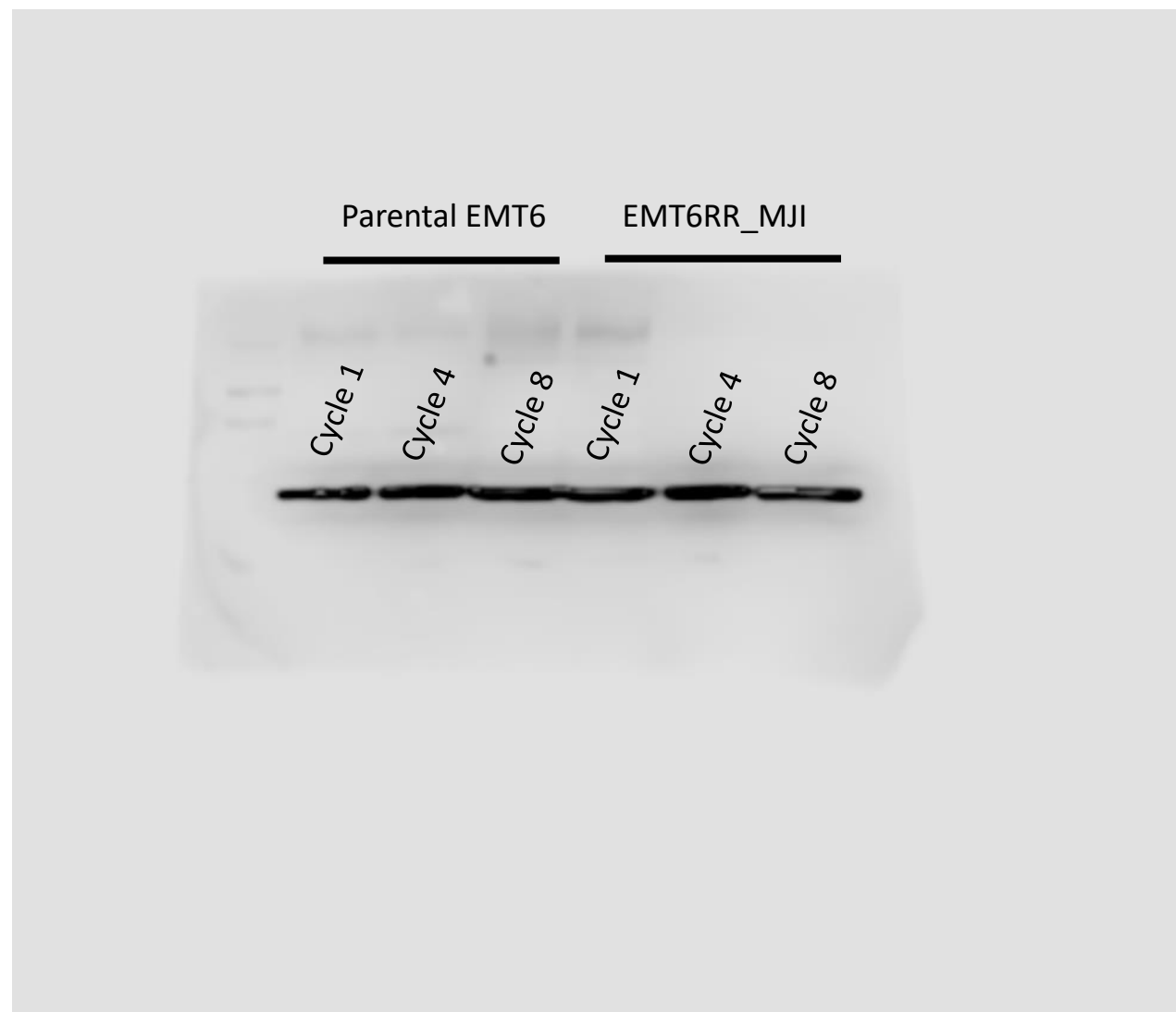

Supplement: Supplementary file 2 — Supplementary Figure 2. [file 41598_2023_29925_MOESM2_ESM.pdf]

## PD1 BAND PROTEIN

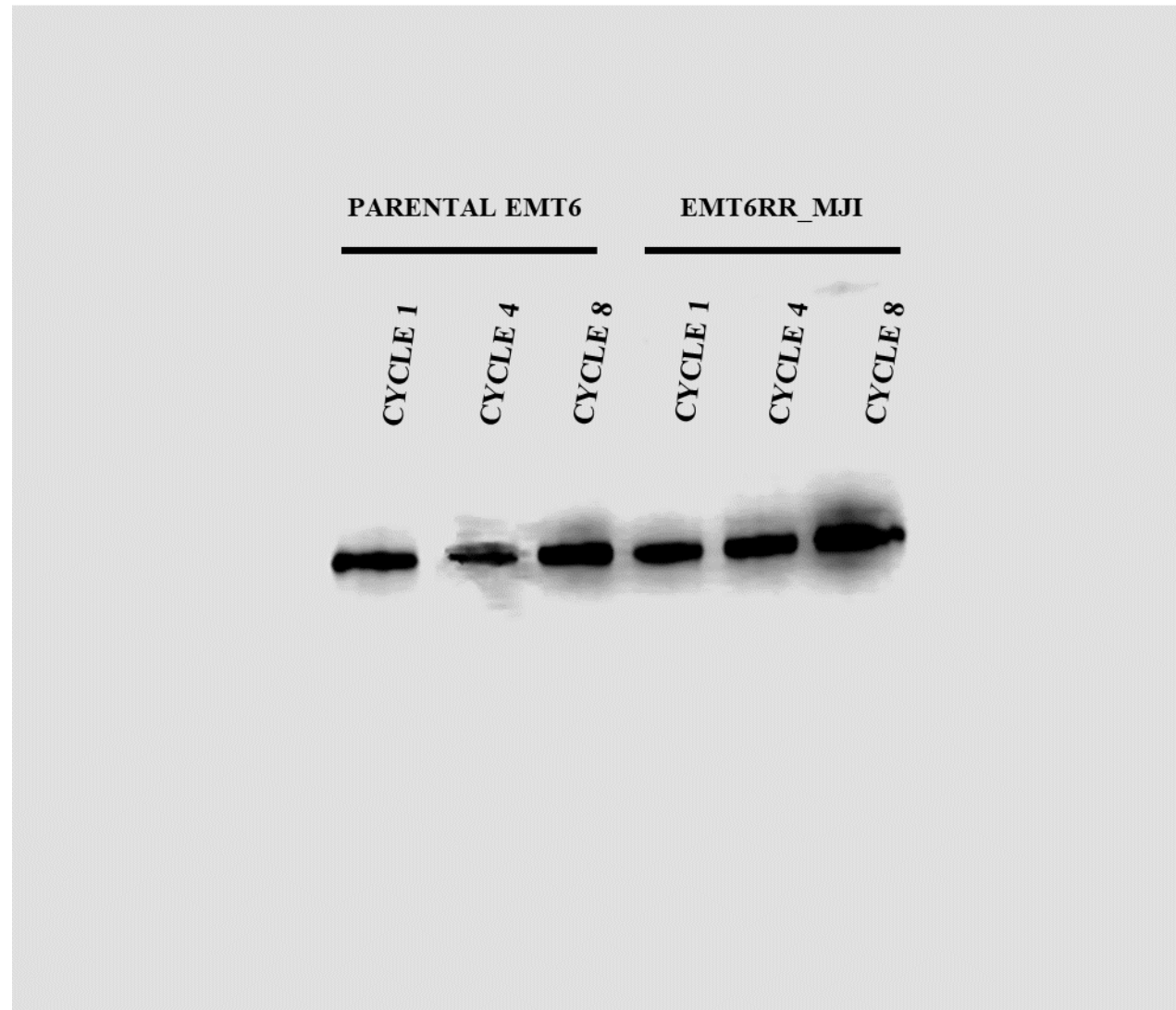

**CTLA4 BAND PROTEIN**

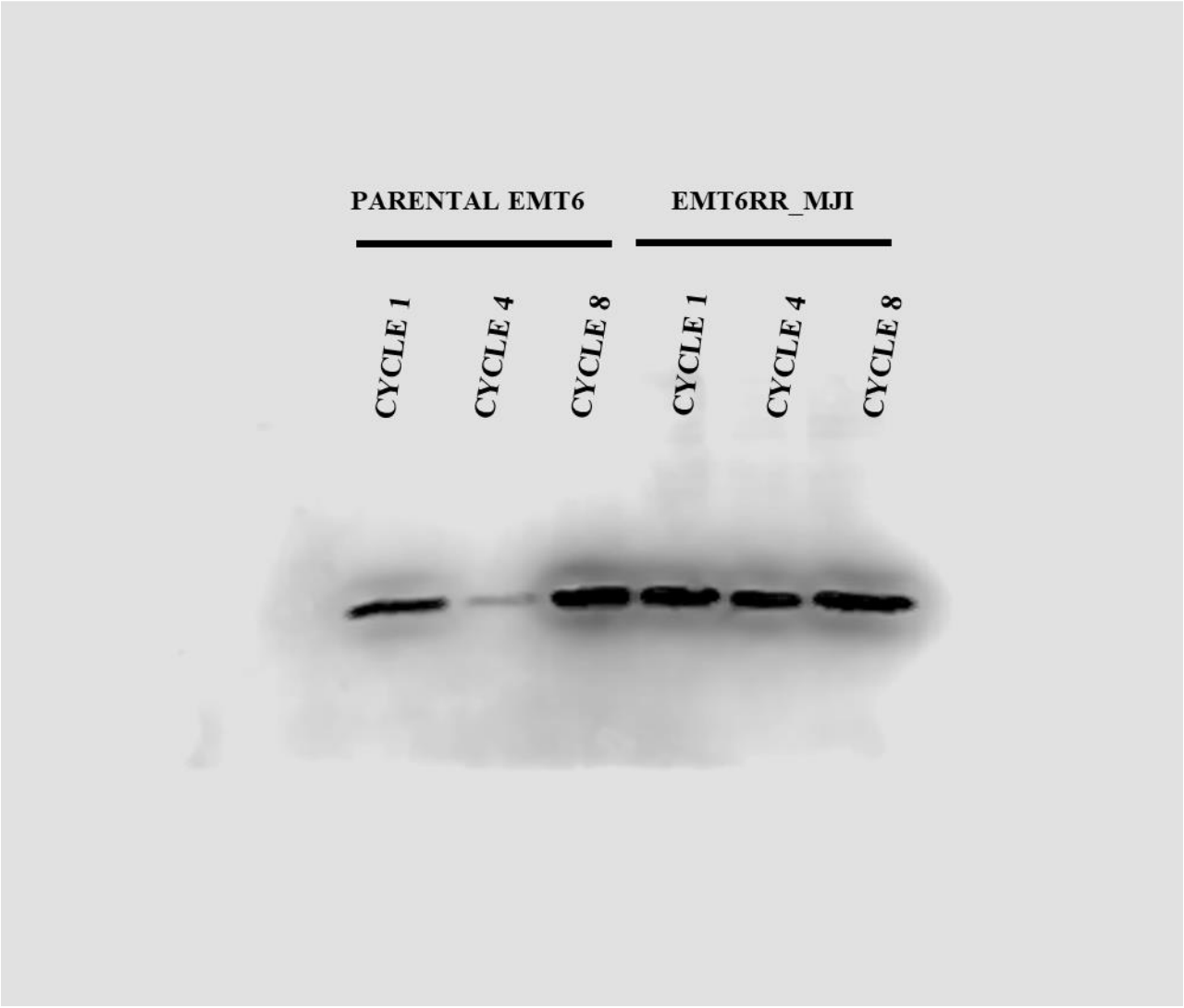

Supplement: Supplementary file 3 — Supplementary Figure 3. [file 41598_2023_29925_MOESM3_ESM.pdf]
