# Supplementary material for: Acquired radioresistance in EMT6 mouse mammary carcinoma cell line is mediated by CTLA-4 and PD-1 through JAK/STAT/PI3K pathway
Source: Sci Rep. 2023 Feb 22;13:3108. doi: 10.1038/s41598-023-29925-x (PMC9946948; doi:10.1038/s41598-023-29925-x)
Supplement: Supplementary file 4 — Supplementary Table S1. [file 41598_2023_29925_MOESM4_ESM.pdf]

Table S1: Forward and Reverse Primer Sequence for housekeeping genes and selected candidate genes.

NCBI GENE ID	GENES	PRIMER SEQUENCE	
		Forward	Reverse
14433	GAPDH	5′-AGGTCGGTGTGAACGGATTTG-3′	5′- TGTAGACCATGTAGTTGAGGTCA-3′
11461	ACTB-ACTIN	5′-GGCTGTATTCCCCTCCATCG-3′	5′-CCAGTTGGTAACAATGCCATGT-3′
26362	AXL	5′- TGGTGAGGGAGGAGCATGTT-3′	5′-AAAAGAAGGGGAGCTTGCTGA-3′
60533	PD-L1	5′-GCTCCAAAGGACTTGTACGTG-3′	5′-TGATCTGAAGGGCAGCATTTC-3′
12525	CD8A	5′-CCGTTGACCCGCTTTCTGT-3′	5′-CGGCGTCCATTTTCTTTGGAA-3′
19225	COX-2	5′-TTCAACACACTCTATCACTGGC-3′	5′- AGAAGCGTTTGCGGTACTCAT-3′
83430	IL23A	5′- ATGCTGGATTGCAGAGCAGTA-3′	5′- ACGGGGCACATTATTTTTAGTCT-3′
14086	FASCIN	5′-GACTGCGAAGGTCGCTACC-3′	5′- CTGATCGGTCTCTTCATCCTGA-3′
56717	MTOR	5′-ACCGGCACACATTTGAAGAAG-3′	5′- CTCGTTGAGGATCAGCAAGG-3′
18412	P62	5′-AGGATGGGGACTTGGTTGC-3′	5′-TCACAGATCACATTGGGGTGC-3′
67443	LC3II	5′-TTATAGAGCGATACAAGGGGGAG-3′	5′- CGCCGTCTGATTATCTTGATGAG-3′
16010	IGFBP4	5′-GAGCGAACATCCCAACAACAG-3′	5′-TGTCCCCACGATCTTCATCTT-3′
22341	VEGFC	5′-TTTGCCAATCACACTTCCTGC-3′	5′-ACACTGTGGTAATGTTGCTGG-3′
16193	IL6	5′-TAGTCCTTCCTACCCCAATTTCC	5′-TTGGTCCTTAGCCACTCCTTC-3′
240168	RASGRP3	5′-AACGACAGTTACTTGCCCAGA-3′	5′-CCTCAGTCATGCGAATCAAACC-3′
216148	SHC2	5′-CAAGGGCGGCTTCATTCAC-3′	5′-CCGAACGATGTAAGAGACCCC-3′
14456	GAS6	5′-TGCTGGCTTCCGAGTCTTC-3′	5′-CGGGGTCGTTCTCGAACAC-3′
494504	APCDD1	5′-CTTCACGGCGTCCAAGTCAT-3′	5′- GCAAGTTCGGTTCACCAGTC-3′
72293	NKD2	5′-GAGCGGAAGAAACGGACCG-3′	5′-CCTTAGGGTCTCCATTGAGCA-3′
320840	NEGR1	5′-TGATCGTGAACTTTGCGCCTA-3′	5′-GCTGTACTTGGAGGGTTGAGG-3′
11658	ALCAM	5′-GGCAGTGGGTTCGTCATAAAC-3′	5′-ATCGCAGAGACATTCAGGGAG-3′
18049	NGF	5′-CCAGTGAAATTAGGCTCCCTG-3′	5′-CCTTGGCAAAACCTTTATTGGG-3′
17392	MMP3	5′-ACATGGAGACTTTGTCCCTTTTG-3′	5′-TTGGCTGAGTGGTAGAGTCCC
