# Supplementary material for: Acquired radioresistance in EMT6 mouse mammary carcinoma cell line is mediated by CTLA-4 and PD-1 through JAK/STAT/PI3K pathway
Source: Sci Rep. 2023 Feb 22;13:3108. doi: 10.1038/s41598-023-29925-x (PMC9946948; doi:10.1038/s41598-023-29925-x)
Supplement: Supplementary file 5 — Supplementary Table S2. [file 41598_2023_29925_MOESM5_ESM.pdf]

Supplementary Table S2: Reaction mix composition

Reagent	Volume	Final concentration
2x SensiFAST™ SYBR® No-ROX One-Step Mix	10 µL	1x
Forward Primer	0.8 µL	400 nM
Reverse Primer	0.8 µL	400 nM
Reverse transcriptase	0.2 µL	-
RiboSafe RNase Inhibitor	0.4 µL	-
H2O subscript	16 µL	-
RNA Template	4 µL	-
	Final volume of **20 µL**	
