# Supplementary material for: Acquired radioresistance in EMT6 mouse mammary carcinoma cell line is mediated by CTLA-4 and PD-1 through JAK/STAT/PI3K pathway
Source: Sci Rep. 2023 Feb 22;13:3108. doi: 10.1038/s41598-023-29925-x (PMC9946948; doi:10.1038/s41598-023-29925-x)
Supplement: Supplementary file 6 — Supplementary Table S3. [file 41598_2023_29925_MOESM6_ESM.pdf]

Table S3: PCR cycle conditions

Cycles	Temperature	Time	Notes
1	45°C	10 min	Reverse transcription
1	45°C	2 min	Polymerase activation
40	95°C	5 sec	Denaturation
	60°C	10 sec	Annealing
	72°C	5 sec	Extension
